# Anti-Influenza Activity of Marchantins, Macrocyclic Bisbibenzyls Contained in Liverworts

**DOI:** 10.1371/journal.pone.0019825

**Published:** 2011-05-20

**Authors:** Yuma Iwai, Kouki Murakami, Yasuyuki Gomi, Toshihiro Hashimoto, Yoshinori Asakawa, Yoshinobu Okuno, Toyokazu Ishikawa, Dai Hatakeyama, Noriko Echigo, Takashi Kuzuhara

**Affiliations:** 1 Laboratory of Biochemistry, Faculty of Pharmaceutical Sciences, Tokushima Bunri University, Yamashiro-cho, Tokushima, Japan; 2 Research group, Research and Production Technology Division, Kanonji Institute, The Research Foundation for Microbial Diseases of Osaka University, Kanonji, Kagawa, Japan; 3 Laboratory of Pharmacognosy, Faculty of Pharmaceutical Sciences, Tokushima Bunri University, Yamashiro-cho, Tokushima, Japan; 4 Laboratory of Pharmaceutical Chemistry, Faculty of Pharmaceutical Sciences, Tokushima Bunri University, Yamashiro-cho, Tokushima, Japan; University of Cambridge, United Kingdom

## Abstract

The H1N1 influenza A virus of swine-origin caused pandemics throughout the world in 2009 and the highly pathogenic H5N1 avian influenza virus has also caused epidemics in Southeast Asia in recent years. The threat of influenza A thus remains a serious global health issue and novel drugs that target these viruses are highly desirable. Influenza A possesses an endonuclease within its RNA polymerase which comprises PA, PB1 and PB2 subunits. To identify potential new anti-influenza compounds in our current study, we screened 33 different types of phytochemicals using a PA endonuclease inhibition assay *in vitro* and an anti-influenza A virus assay. The marchantins are macrocyclic bisbibenzyls found in liverworts, and plagiochin A and perrottetin F are marchantin-related phytochemicals. We found from our screen that marchantin A, B, E, plagiochin A and perrottetin F inhibit influenza PA endonuclease activity *in vitro*. These compounds have a 3,4-dihydroxyphenethyl group in common, indicating the importance of this moiety for the inhibition of PA endonuclease. Docking simulations of marchantin E with PA endonuclease suggest a putative “fitting and chelating model” as the mechanism underlying PA endonuclease inhibition. The docking amino acids are well conserved between influenza A and B. In a cultured cell system, marchantin E was further found to inhibit the growth of both H3N2 and H1N1 influenza A viruses, and marchantin A, E and perrotein F showed inhibitory properties towards the growth of influenza B. These marchantins also decreased the viral infectivity titer, with marchantin E showing the strongest activity in this assay. We additionally identified a chemical group that is conserved among different anti-influenza chemicals including marchantins, green tea catechins and dihydroxy phenethylphenylphthalimides. Our present results indicate that marchantins are candidate anti-influenza drugs and demonstrate the utility of the PA endonuclease assay in the screening of phytochemicals for anti-influenza characteristics.

## Introduction

An influenza A pandemic in 1918, known as the Spanish flu, caused 50 million deaths worldwide [1]–[3]. More recently, the avian H5N1 influenza A virus, which is highly pathogenic to humans, caused epidemics in Southeast Asia [4] and a new strain (Pandemic (H1N1) 2009) that emerged from pigs into humans caused a further pandemic in 2009 [5], [6]. Strategies are thus needed to prevent future expansions of these viruses which remain a serious global health issue. Although neuraminidase inhibitors such as oseltamivir are widely used as anti-influenza drugs [7], [8], some side effects of these agents and also the emergence of viral strains that are resistant to these agents have now been reported [9]–[11]. For the future prevention and control of influenza outbreaks, it will therefore be critical to develop novel drugs that are not based on neuraminidase inhibition.

The influenza A genome consists of segmented single stranded RNA (-) and its transcription and replication require the activity of a highly conserved RNA-dependent RNA polymerase [12]–[14]. The influenza A RNA-dependent RNA polymerase is composed of three subunits, PA, PB1 and PB2, and synthesizes viral mRNAs using short capped primers derived from host cellular mRNAs that are cleaved by the viral endonuclease [12], [13]. Yuan *et al*. and Dias *et al*. have shown previously that the N-terminal domain of the PA subunit contains a typical endonuclease active site and also harbors RNA/DNA endonuclease activity [15]–[17]. This domain is essential for viral growth and we speculated that it would be a highly effective target domain to which novel anti-influenza A drugs would bind.

**Figure 1:**
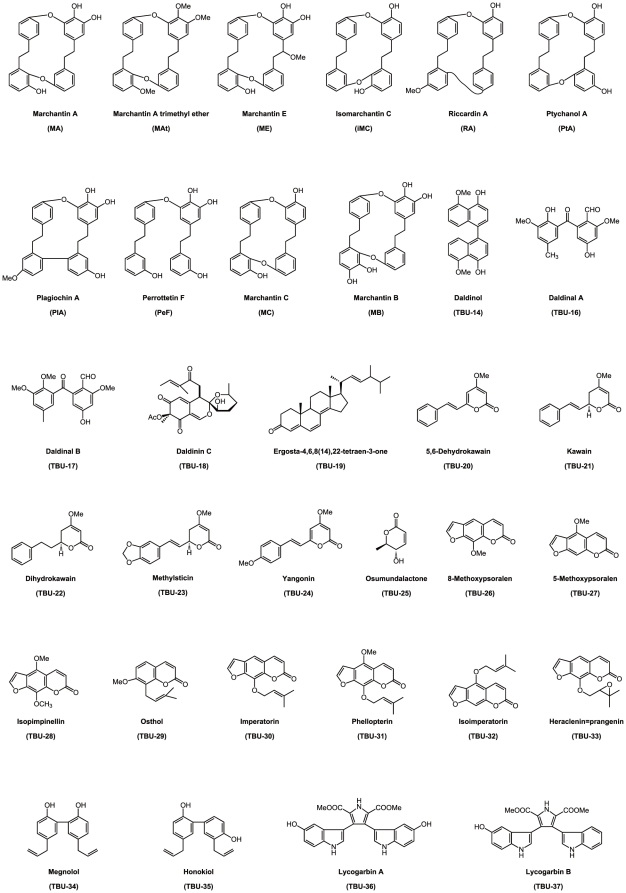
Chemical structures of the phytochemicals tested in this study. Diverse chemical structures of the phytochemicals screened in this study for their anti-influenza A activities. The sources for these structures are described in the materials and methods.

The Marchantiophyta liverworts and several other types produce a large number of various lipophilic terpenoids, sesqui- and diterpenoids, phenolics [18]–[28], bibenzyls and bisbibenzyls [21]–[28]. Marchantin (Fig. 1), a unique macrocyclic structure denoted as a cyclic bisbibenzyl, is a phytochemical found in several liverworts. The yield of marchantin A (MA) is extremely high (a 100–120 gram quantity can be obtained from 2 kg of dried material), for the Japanese *Marchantia poleacea* var. *diptera*. Marchantins A (MA), B (MB), C (MC) and E (ME) and isomarchantin C (IMC) from *Marchantia polymorpha* and *M. paleacea*, riccarrdin A (RA) from *Riccardia multifida*, ptychantol A (PtA) from *Ptychanthus striatus*, plagiochin A (PlA) from *Plagiochila fruticosa*, all of which are marchantin-like phytochemicals, have one or two diaryl ether or biphenyl bonds (Fig. 1). The bisbibenzyl, perrottetin F (PeF), isolated from *Lunularia cruciata* is also similar to marchantin but is not cyclic (Fig. 1) [18]–[23]. These compounds have shown interesting biological activities in the past such as cytotoxicity, 5-lipoxygenase inhibitory activity, inhibitory activity against NO production stimulated by lipopolysaccharide (LPS), antimicrobial activity, antitumor activity, and farnesoid X receptor (FXR)-activating activity, among others [24], [25].

In our current study, we screened 33 different phytochemicals using PA endonuclease *in vitro* and then examined the anti-influenza activities of these selected compounds. Marchantin and other phytochemicals (Fig. 1) were purified as previously described [18]–[28]. We performed initial *in vitro* screenings for the inhibition of PA endonuclease activity. After this *in vitro* selection, we performed an anti-virus assay. We found from these analyses that marchantins and related chemicals inhibit PA endonuclease activity, and exert anti-influenza activity in cultured cells and in focus formation assays.

## Results

### Inhibition of PA endonuclease by marchantins

**Figure 2:**
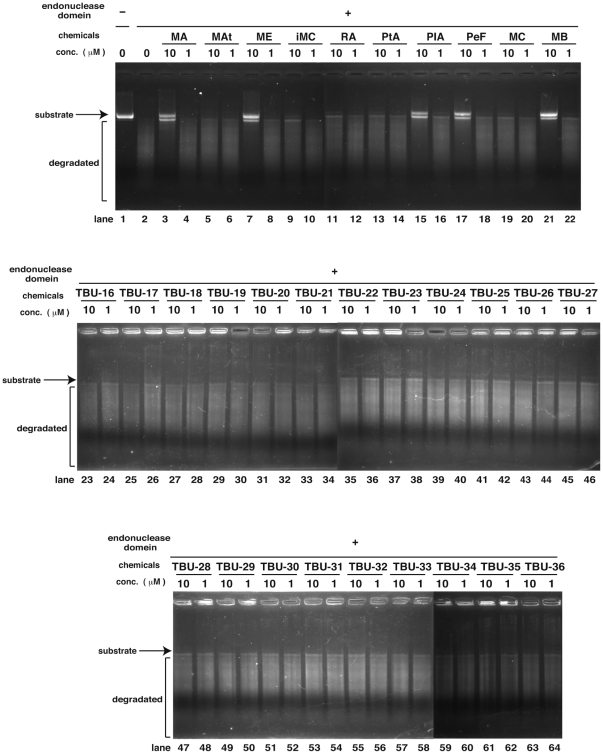
Screening of anti-influenza phytochemicals using a PA endonuclease-inhibition assay. Screening of phytochemicals for anti-influenza A activity using a PA endonuclease assay. The effects of the various phytochemicals upon the endonuclease activity of the PA N-terminal domain of the influenza A RNA polymerase were tested. The recombinant PA N-terminal domain protein was added to each reaction at 0.35 µg/ 100 µl. A zero control (no PA domain added) was also assayed. Phytochemicals were added at a 1 or 10 µM dose and M13mp18 was used as the substrate.

We tested 33 phytochemicals in a PA endonuclease-inhibition assay, as shown in Figure 1, using the recombinant PA endonuclease domain protein. In this assay, we incubated 0.1 µM of recombinant PA endonuclease domain with both 1 or 10 µM of each phytochemical. The PA endonuclease domain digests circular single stranded DNA *in vitro* (Fig. 2, lanes 1 & 2) [16], [29] and we examined whether any of the phytochemicals in our panel could inhibit this activity. Only marchantin A (MA), B (MB), E (ME) and the marchantin-related chemicals, perrottetin F (PeF) and plagiochin A (PlA), showed any inhibitory activity at a 10 µM concentration (Fig. 2, lanes 3, 7, 15, 17 & 21). This is the first evidence that the phytochemicals derived from the liverwort plant can inhibit the influenza A endonuclease. Each of the five inhibitory compounds also contain a dihydroxyphenethyl group (Fig. 1), which is absent from the other chemicals in the study panel (Fig. 1), thus suggesting its importance for PA endonuclease inhibition.

### Docking simulation for marchantin with the influenza PA endonuclease domain

**Figure 3:**
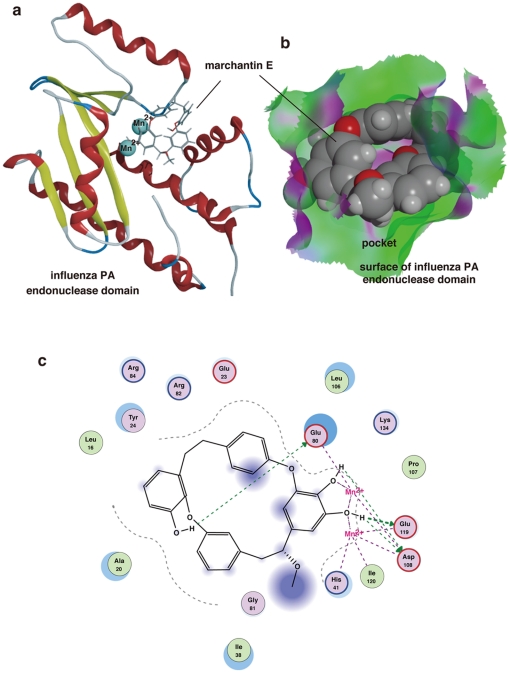
Docking simulation of marchantin E with influenza PA endonuclease. **A)** Docking simulation analysis of marchantin E with the PA endonuclease domain of the influenza A RNA polymerase. PA endonuclease is depicted as a ribbon structure. The α-helix and β-strands are shown in red and yellow, respectively. Manganese ions in the PA endonuclease are shown as cyan. Marchantin E is shown as a stick structure with oxygen atoms indicated in red. **B)** The fitting of marchantin E to the active pocket of the PA endonuclease. Marchantin E is displayed in a sphere mode. The surface of the pocket of the PA endonuclease is indicated in green and purple. The grey and red balls indicate carbon and oxygen atoms in marchantin E, respectively. **C)** Two dimensional analysis of the interaction between marchantin E and PA endonuclease. The chemical structure of marchantin E is shown in the center with the key interacting amino acids shown around it. The dihydroxyphenyl group of marchantin E interacts with two manganese ions. The hydroxy groups of marchantin bind to amino acids in PA endonuclease.

To further investigate how marchantin and its related chemicals inhibit PA endonuclease activity and why the dihydroxyphenethyl group is important for this function, we performed *in silico* docking simulation analysis of marchantin E with PA endonuclease using tertiary structure information. The tertiary structure of PA endonuclease has already been resolved [30] and the tertiary structure and flexibility of marchantin E was determined in our present study using the MOE program [31]–[33]. The analysis indicated that marchantin fits well into the active pocket of PA endonuclease (Fig. 3A, B). PA endonuclease harbors two Mn^2+^ ions in its active site (Fig. 3A) [30] which are important for its activity [15], [16]. Our docking simulation experiments further revealed that the dihydroxy group in the dihydroxyphenethyl group of marchantin E chelates the Mn^2+^ ions within PA endonuclease (Fig. 3C). This dihydroxy group also interacts with several active amino acids (Fig. 3C). The aromatic rings and methoxy group of marchantin E bind to the active pocket of PA endonuclease through a hydrophobic interaction (Fig. 3C). We further performed docking simulation analysis of marchantin A with PA endonuclease and the results were almost identical. This docking analysis is consistent with our observations of endonuclease inhibition, and confirms the importance of the dihydroxyphenethyl group.

### Inhibition of influenza A and B viruses by marchantins

**Figure 4:**
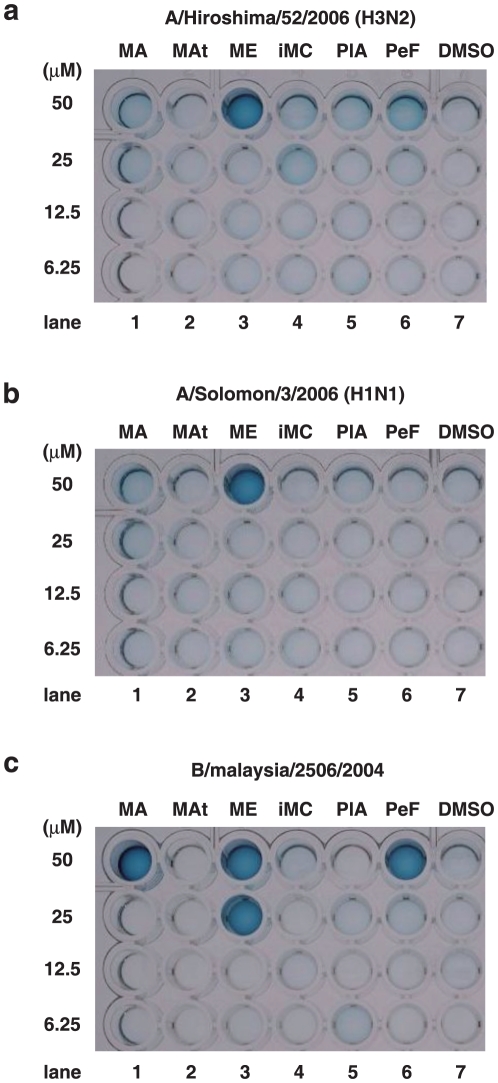
Anti-influenza activity of marchantins. Inhibition of the growth of influenza A or B viral by phytochemicals. MDCK cells were treated with influenza A/Hiroshima/52/2006 (H3N2) (**A**), A/Solomon/3/2006 (H1N1) (**B**) or B/Malaysia/2506/2004 (**C**) viruses. The cells were then treated with various concentrations of phytochemicals (6.25–50 µM), some of which suppressed viral-induced cell death. DMSO was used as the phytochemical solvent.

Following *in vitro* selection using the endonuclease-inhibition assay, as a second screening, we next examined the anti-viral activity of MA, ME, perrottetin F and plagiochin A against the influenza strains A/Hiroshima/52/2006 (H3N2), A/Solomon/3/2006 (H1N1) and B/Malaysia/2506/2004. As a control, two phytochemicals, marchantin A trimethoxy and isomarchantin C, which do not inhibit PA endonuclease, were also examined. The phytochemicals and viruses were mixed and then added to cultures of Madin-Darby canine kidney (MDCK) cells [34]. As shown in Figure 4, the blue color indicates viable cells, indicating that the influenza virus had been inhibited [35]. Marchantin E showed the strongest anti-influenza activity against the A/Hiroshima/52/2006 (H3N2) and A/Solomon/3/2006 (H1N1) viruses at a 50 µM concentration (Fig. 4A, B), and against B/Malaysia/2506/2004 virus at a 25 µM dose (Fig. 4C). Marchantin A and perrottetin F were found to have inhibitory activity against the B/Malaysia/2506/2004 virus at 50 µM (Fig. 4). The other compounds and DMSO solvent alone (negative control) did not show anti-influenza activity. All of the inhibitory phytochemicals also showed PA endonuclease-inhibition activity and harbor a dihydroxyphenethyl group within their structures. This demonstrates that the *in vitro* PA endonuclease activity assay is a useful tool for the screening of potential anti-influenza chemicals and also confirms the importance of the dihydroxyphenethyl group for anti-influenza activity.

**Figure 5:**
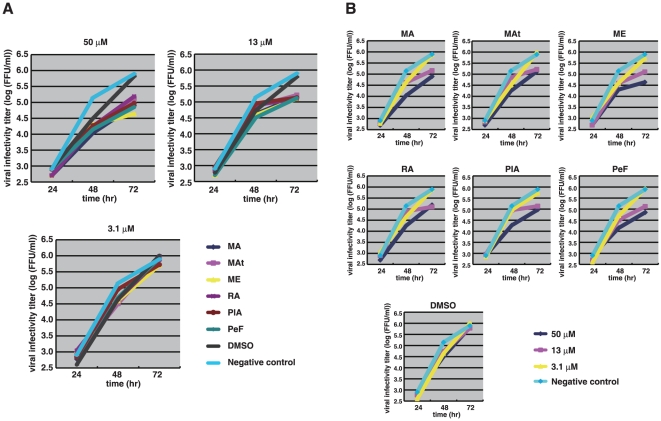
Decrease of the viral infectivity titer by marchantins. Decrease of viral infectivity titer of influenza B viral by phytochemicals. MDCK cells were treated with influenza B/Malashia/2506/2004 virus. The cells were then treated with various concentrations of phytochemicals (3.1–50 µM), some of which decreased viral infectivity titer. DMSO was used as the phytochemicals solvent. (**A**) Viral growth curve under exposure to various concentrations of marchantins. The horizontal axis is the culture time and the vertical axis is the logarithm of virus infectivity titer (focus formation unit (FFU)). (**B**) Viral growth curve in the presence of marchantins and comparisons of the same concentration. The horizontal axis is the culture time and the vertical axis is the logarithm of virus infectivity titer (focus formation unit (FFU)).

To examine whether marchantins can decrease the viral infectivity titer or not, we performed a focus-formation assay [36] without pre-incubation prior to infection. This assay determines viral growth in cells and can exclude the possibility of virus-absorption effects. We counted the viral foci using the PAP (peroxidase anti-peroxidase) method along with time course. The raw data are shown in Table S1 and the results are summarized in Table 1 and Figure 5. The marchantins and related chemicals decreased viral focus-formation in a dose-dependent manner, indicating that these compounds inhibit viral growth in host cells. Among these chemicals, MA, ME and PeF showed the strongest activity at 50 µM, as shown in Table 1 (highlighted in blue). MA, ME and PeF each contain a dihydroxyphenethyl group, again confirming the importance of this moiety. Marchantin E showed the strongest activity (Table 1). These results are consistent with the data shown in Figure 4.

**Table 1:** Specific activity of marchantins against viral infectivity titer.

Chemicals	Conc. (µM)	Log (Focus formation unit/ml)			Dihydroxy phenethyl group
		24 (hr)	48 (hr)	72 (hr)	
MA	50	2.7	4.04	4.9	+
	13	2.85	4.62	5.16	
	3.1	2.78	4.64	5.87	
MAt	50	2.7	4.24	5.12	-
	13	2.85	4.86	5.22	
	3.1	2.9	4.5	5.97	
ME	50	2.7	4.3	4.64	+
	13	2.7	4.59	5.1	
	3.1	3.04	4.6	5.71	
RA	50	2.7	4.25	5.19	-
	13	2.9	4.9	5.1	
	3.1	3.04	4.65	5.88	
PlA	50	2.9	4.27	4.97	+
	13	2.95	4.95	5.13	
	3.1	2.85	4.96	5.72	
PeF	50	2.9	4.15	4.85	+
	13	2.7	4.51	5.12	
	3.1	2.6	4.7	5.92	
DMSO (solvent)	50	2.9	4.5	5.82	
	13	2.78	4.73	5.79	
	3.1	2.6	4.64	6	
Negative control	-	2.9	5.15	5.89	

### Conserved amino acids important for docking between influenza A and B

**Figure 6:**
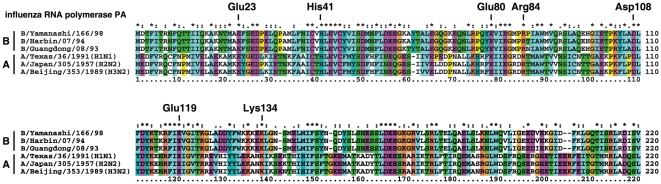
Docking amino acids are conserved between influenza A and B. The docking amino acids (Glu23, His41, Glu80, Arg84, Asp108, Glu119 and Lys134) identified in Figure 3 are shown in an alignment of influenza A and B amino acid sequences. This alignment was generated using the clustal X program.

Since marchantin E inhibits the growth of both influenza A and B (Fig. 4), we predicted that the amino acids in PA which are important for binding to marchantin E should be conserved between these virus strains. Using the clustal X program [37], we compared the PA amino acid sequences of influenza A and B (Fig. 6). Glu23, His41, Glu80, Arg84, Asp108, Glu119 and Lys134 are the predicted docking amino acids as shown in Figure 2 and are fully conserved (Fig. 6). The endonuclease-inhibition, anti-influenza activity and docking simulation results are thus highly consistent.

### Comparisons between marchantin and other inhibitors of influenza A

**Figure 7:**
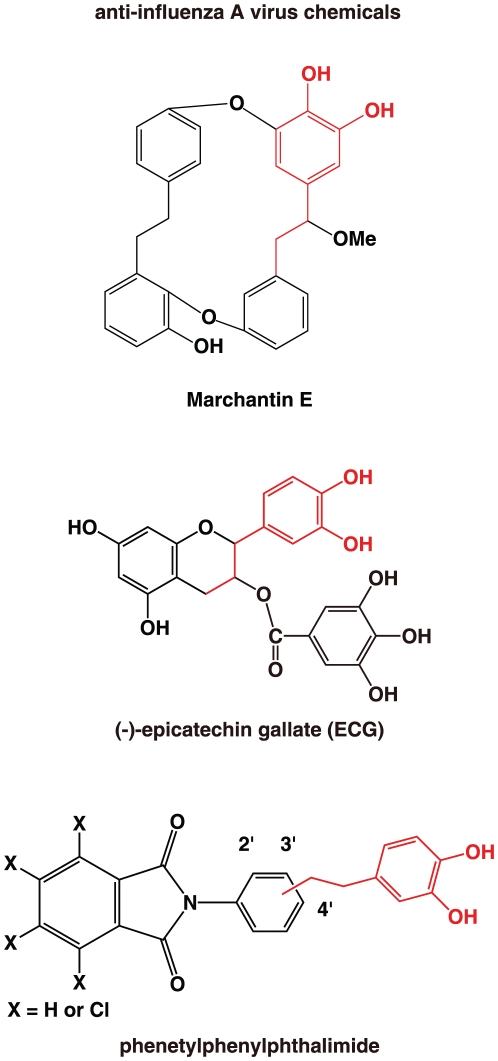
Consensus structure of chemicals with anti-influenza activity. Consensus structure of the phytochemicals and synthesized chemicals possessing anti-influenza A activity. The dihydroxyphenethyl group (shown as red) is common to marchantin, catechin and several phthalimide molecules which all inhibit the influenza virus.

**Figure 8:**
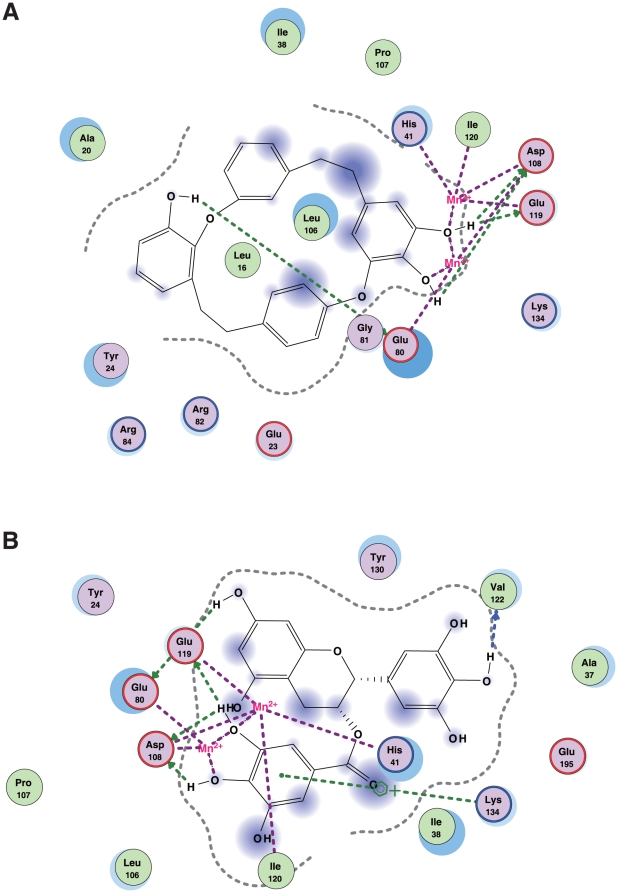
Comparison between marchantins and catechins that bind to PA endonuclease. Docking simulation analysis of marchantin A (panel A) or EGCG (panel B) with the PA endonuclease domain. Two dimensional analysis of the interactions of marchantin A or EGCG with PA endonuclease is shown. The chemical structures of marchantin A and EGCG is shown in the center. The interacting amino acids of PA endonuclease are shown around them. The dihydroxyphenyl groups of both marchantin A and EGCG interact with two manganese ions.

To determine whether a common active chemical group is contained among inhibitors of the influenza virus, we compared marchantin with other influenza and PA endonuclease inhibitors. Previously, we have reported that the green tea catechins, epigallocatechin gallate (EGCG) and epicatechin gallate (ECG) [38], [39] have these inhibitory properties [17] (Fig. 7). We have also reported that several synthesized phthalimide chemicals, namely N-{2-[2-(3,4-dihydroxyphenyl)ethyl]phenyl}phthalimide and N-{3-[2-(3,4-dihydroxyphenyl)ethyl]phenyl}phthalimide also inhibit PA endonuclease and have anti-influenza activity [29] (Fig. 7). As marchantins and catechins are natural products, whereas the phenethylphenylphthalimides are synthetic, their structures are quite different (Fig. 7). Significantly however, all contain a dihydroxyphenethyl group (Fig. 7), again confirming its importance in conferring anti-influenza activity. Docking simulation analyses of marchantin A or EGCG with PA endonuclease showed that both these molecules chelate divalent manganese ions within PA endonuclease via dihydroxy groups (Fig. 8). These simulation analyses further underlie the importance of the dihydroxyphenethyl groups that are common to anti-influenza chemicals.

## Discussion

This is the first report to show that secondary metabolites from liverworts have anti-influenza activity. Marchantin has a unique macrocyclic structure, and is therefore a novel type of anti-influenza chemical. Whilst marchantin targets PA endonuclease, oseltamivir does so for neuraminidase. We speculated that a combination of marchantin and oseltamivir would be effective against influenza, because it can inhibit two independent steps that are essential for the growth of this virus. We believe therefore that a marchantin solution or a liverwort extract would likely be an efficient gargle for the prevention of influenza.

As shown in Figures 3c and 8, the amino acids in PA that are important for docking with marchantin A or E are common (also see Fig. 8). We speculate that the small differences in the docking mode between marchantin A and E that we observed may be due to the methoxy group of marchantin E. Based on our present analyses, we propose a “fitting and chelating model” as the mechanism by which marchantin and its related chemicals inhibit the influenza PA endonuclease. These data thus represent an important advance for future strategies to refine marchantin-based drug design using an *in silico* binding simulation with PA. Our structure-function relationship analysis and docking simulation analysis also provide valuable new information for the design of novel anti-influenza drugs.

## Materials and Methods

### Purification of phytochemicals

Marchantins were isolated from the methanol extracts of *Marchantia polymorpha* and *M. paleacea* var. *diptera* [19], [24], perrottetin F from *Plagiochila sciophila*, and plagiochin A from *Radula kojana*. Daldinol, daldinals A and B, daldinin C and ergosta-4,6,8 (14), 22-tetraen-3-one were isolated from the fungus *Daldinia concentrica* [40], [41]. 5, 6-Dehydrokawain, kawain, dihydrokawain, methysticin and yangonin were isolated from Fijian Kawa (*Piper methysticum*) [42]. Osumundalactone was isolated from the fungus *Paxillus atromentosus* [43]. 8-Methoxypsoralen, 5-methoxypsoralen, isopimpinellin, osthol and imperatprin were isolated from the fruits of *Cnidium monnieri*, belonging to the Umbelliferae. Phellopterin, isoimperatorin and heraclenin (also known as pragenin) were isolated from the root of *Angelica dahurica*, also belonging to the Umbelliferae. Magnolol and honokiol were isolated from the bark of the *Magnolia obovata* belonging to the Magnoliaceae. Lycogarbins A and B were isolated from the slime molds (Myxomycete) *Lycogala epidendrum* [44].

### Expression and purification of the PA endonuclease domain

The influenza (A/PR/8/34) H1N1 RNA polymerase PA plasmid, pBMSA-PA, was sourced from the DNA Bank, Riken BioResource Center (Tsukuba, Japan; originally deposited by Dr. Susumu Nakada) [45]. The cDNA fragment corresponding to the PA N-terminal endonuclease domain (residues 1–220) was amplified by PCR [46] from pBMSA-PA using the primers PA endonuclease forward *Nde*I, GCC GTT CAT ATG GAA GAT TTT GTG CGA CAA and PA endonuclease reverse *Bam*HI, GCC GTT GGA TCC TAT TGG TCG GCA AGC TTG CG. The amplified product was then subcloned into the pET28a(+) plasmid (Novagen, Madison, WI) at the *Nde*I and *Bam*HI restriction sites. The resulting construct was then introduced into BL21-CodonPlus *E. coli* cells (Stratagene, La Jolla, CA). The induction of 6x his-tagged recombinant protein expression from these constructs was achieved by the addition of isopropyl -D-thiogalactopyranoside (IPTG) [47] to the bacterial cultures in TBG-M9 medium and this was followed by purification using Ni^2+^-agarose [48]. The recombinant PA endonuclease domain protein was further purified to near homogeneity using a HiTrap™ Q FF column (GE Healthcare, Buckinghamshire, UK) with the AKTA™ prime plus system (GE Healthcare).

### PA endonuclease activity assay

Influenza A RNA polymerase PA endonuclease activity assays were performed essentially as described by Dias *et al*. [14]–[16] with some modifications. Briefly, the pH conditions were modified from 8.0 to 7.3 and 1 µg of M13mp18 single stranded circular phage DNA was used as the assay substrate. A total of 0.35 µg of recombinant PA endonuclease domain was added to 100 µl of assay buffer in each reaction (the final concentration of the protein was about 0.1 µM). Phytochemicals (summarized in Fig. 1) were then added to the reaction and products were analyzed by agarose electrophoresis and stained with ethidium bromide.

### Inhibition of viral growth

Madin-Darby canine kidney (MDCK) cells [34] were cultured in MEM (minimum essential medium; Gibco/Invitrogen, Carlsbad, CA) supplemented with 10% fetal bovine serum in a 5% CO_2_ incubator at 37 °C. A confluent monolayer of MDCK cells was prepared in each well of a 96 well plate. Various concentrations (6.25–50 µM) of phytochemicals were then mixed with TCID_50_ (fifty-percent of the infectious dose) of A/Hiroshima/52/2006 (H3N2), A/Solomon/3/2006 (H1N1) or B/Malashia/2506/2004 influenza strains and incubated at 37°C for 30 min [35]. The MDCK cells were then washed with PBS(-) and the viral mixture was added to the cells. Treated cells were then incubated for four days at 34°C under 5% CO_2_. After incubation, the medium was removed and cells were fixed with a 10% formaldehyde solution. Viable cells were stained with NB solution (0.1% napthol blue black, 0.1% sodium acetate and 9% acetic acid) [35].

### Focus-formation assay

MDCK cells were incubated on micro plates (24-well) at 37 °C for three days in a CO_2_ incubator. The cells were washed with PBS(-) and influenza virus (B/Malaysia/2506/2004) solution was added at an m.o.i of 0.001. Viruses were absorbed on cells at 37 °C for 30 min and the cells were washed by PBS(-) to remove unabsorbed virus. Concentrations of 50, 12.5 or 3.1 µM of chemicals in growth medium were added into each well. The cells were then incubated at 37°C for three days and a portion of the supernatant was sampled each day. The sampled supernatants were subjected to a focus forming unit (FFU) assay using the PAP (rabbit peroxidase anti-peroxidase) method. Fresh MDCK cells were incubated in micro plates (96-well) at 37°C for three days. The obtained virus solutions were 10-fold titrated in micro plates (from 10 to 10^8^ times). The cells were then washed with PBS(-) and 100 µl of the titrated virus solutions was added to the cells. Viruses were absorbed on the cells at 37°C for 60 min with shaking at 15 min intervals. The cells were washed with PBS(-) to remove unabsorbed virus and MEM medium (serum (-)) was then added, followed by an incubation at 37 °C for 1 day. Supernatant was removed and cells were fixed with ethanol for 10 min at room temperature. The ethanol was then removed and cells were dried.

The staining of foci via the PAP method was performed as follows: 50 µl of a 1000 fold-dilution of the primary antibody (mouse anti-influenza A or B NP serum) was added to each well and incubated at 37 °C for 30 min. Each well was washed with PBS(-). 50 µl of a 1∶1000 dilution of secondary antibody (rabbit anti-mouse IgG serum) was then added into each well and incubated at 37 °C for 30 min. Each well was further washed in PBS(-). 50 µl of a 1∶500 dilution of a third antibody (goat anti-rabbit IgG serum) was next added into each well and the cells were treated as before. Finally, 50 µl of a 1∶10000 PAP complex solution was added to each well and incubated at 37°C for 30 min. Each well was washed by PBS(-). This was followed by the addition of 50 µl of substrate (3,3′-diaminobenzidin tetrahydrochloride) for 10–15 min at room temperature, washing with water and drying. The infectivity titer (FFU) was calculated by counting the numbers of foci i.e. numbers of foci per well x titration of virus solution x 1000 / 100 (volume of virus solution) = infectivity titer (FFU/ml).

### *In silico* docking simulation analyses of marchantins and catechin with the PA endonuclease domain of influenza A

All molecular modeling studies were performed using Molecular Operating Environment (MOE; Chemical Computing Group, Quebec, Canada) software [31]–[33]. The X-ray crystallographic structure of the endonuclease domain of PA subunit of influenza A virus RNA dependent RNA polymerase (PDB ID: 3HW5) was obtained from a protein data bank [30]. This enzyme was prepared for docking studies in which (i) the ligand molecule was removed from the enzyme active site; (ii) hydrogen atoms were added to the structure with a standard geometry; (iii) the structure was minimized using a MMFF94s force-field; (iv) MOE Alpha Site Finder was used for active site searches within the enzyme structure and dummy atoms were created from the obtained alpha spheres; and (v) the obtained model was then used in the ASEDock program (Ryoka Systems Inc., Tokyo, Japan). A total of 250 conformations of marchantin A or E were generated by Low Mode MD. The used parameters in step 1 were as follows: cut off value, 4.5; RMS gradient, 10; and energy threshold, 500. The used parameters in step 2 (optimization) were as follows: cut off value,8; RMS gradient, 0.1.

## Supporting Information

Table S1 **Effect of marchantins on viral infectivity titer (raw data).** We tested the effects of marchantins on viral infectivity-titer and counted the viral foci along with time course. This raw data are calculated and summarized in Table 1 and presented as graphs in Figure 5.
(XLS)
